# Supplementary material for: An interhemispheric neural circuit allowing binocular integration in the optic tectum
Source: Nat Commun. 2019 Nov 29;10:5471. doi: 10.1038/s41467-019-13484-9 (PMC6884480; doi:10.1038/s41467-019-13484-9)
Supplement: Supplementary file 1 — Supplementary Information [file 41467_2019_13484_MOESM1_ESM.pdf]

Supplementary Information

An interhemispheric neural circuit allowing binocular integration in the optic tectum

(Gebhardt et al.)

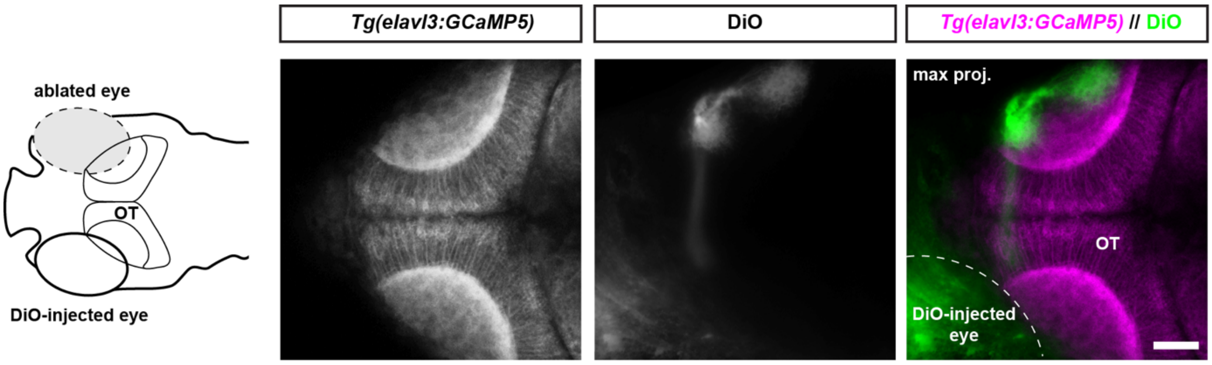

Supplementary Fig. 1

Supplementary Fig. 1. Eye enucleation.

Monocular ablation at 3 dpf does not induce re-routing of retinal fibres of the remaining eye to the ipsilateral tectal hemisphere by 5 dpf as shown by anterograde labelling of retinal ganglion cell axons with DiO (pseudo-coloured in green). The optic tectum is labelled by the pan-neuronal transgene *Tg(elavl3:GCaMP5G)* (pseudo-coloured in magenta). Scale bar = 50 μm.

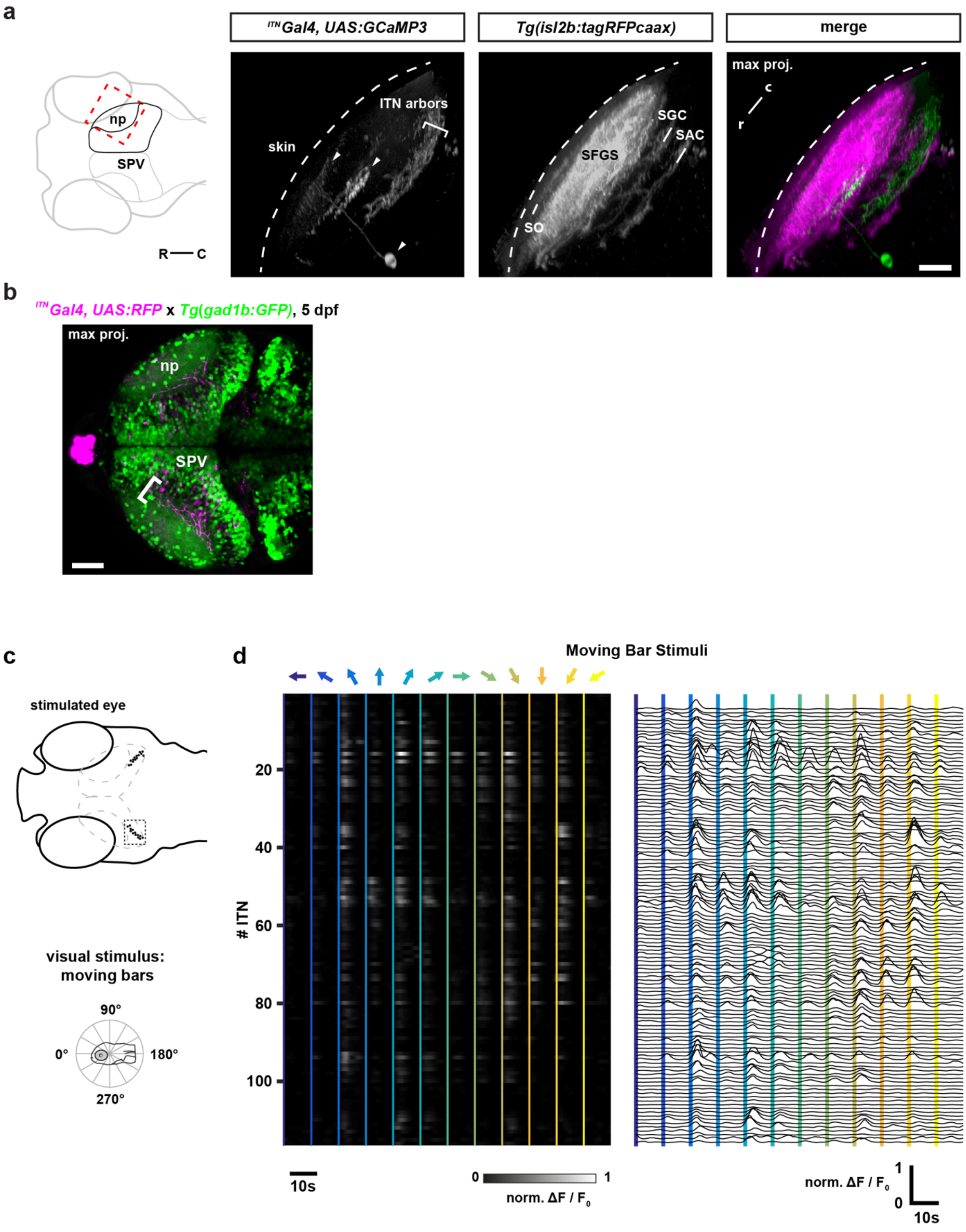

Supplementary Fig. 2

Supplementary Fig. 2. ITNs arborize in the deep layers of the tectal neuropil and respond to visual stimuli.

(a) Maximum intensity projection of the right tectal neuropil viewed dorsally of a representative 3 dpf *ITNGal4, UAS:GCaMP3*, *Tg(isl2b:tagRFPcaax* larva*)*. ITN arbours are exclusively observed in the deep layers of the tectal neuropil in between the SGC and SAC layer. Occasionally, single PVNs are labelled by the *ITNGal4* transgene but PVN neurites are rarely overlapping with ITN arbours and can be easily distinguished based on their morphology. A single bi-stratified periventricular neuron in the SPV is labelled in this particular ITNGal4 larva (white arrows pointing at the PVN's cell body and its arbours) and was used as anatomical reference. Retinal arbours labelled by the *isl2b* promotor are pseudo-coloured in magenta. Scale bar = 20 µm. (R: rostral, C: caudal, np: tectal neuropil, SO: *stratum opticum*, SFGS: *stratum fibrosum et griseum superficiale*, SGC: *stratum griseum centrale*, SAC: *stratum album centrale*, SPV: *stratum periventricuare*).

(b) ITN arbours are predominantly found in the deep layers of the tectal neuropil (most likely between the SAC and SGC in the tectal neuropil, indicated by the white bracket). This is consistent with the location of visually-evoked activity observed in the ipsilateral deep neuropil after monocular enucleation (see Fig. 1d). Single PVNs were also labelled by the *ITNGal4* transgene but PVN neurites were mostly non-overlapping with ITN arbours and could thus be easily distinguished based on their morphology. Scale bar = 50 µm. (np: tectal neuropil, SPV: *stratum periventricuare*).

(**c**) 5 dpf larvae expressing GCaMP3 in ITNs were imaged with a 2-photon microscope while visually stimulating the contralateral eye with moving bars (bar width: 9 °, speed: 20 °/s, direction: randomly chosen for each stimulus epoch from 12 angular directions 30 ° apart) running across the field of view.

(**d**) ITNs responded strongly to visual motion stimuli (n = 116 ITNs from 14 larvae). Vertical lines indicate the begin of a stimulus interval color-coded according to the stimulus direction. Source data are provided as a Source Data file.

a

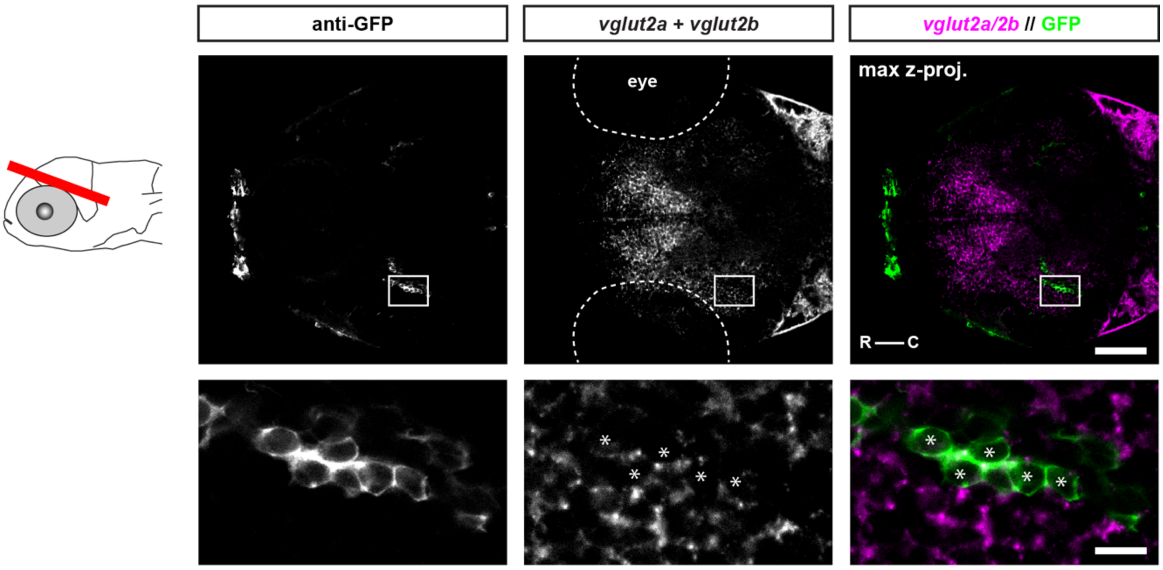

b

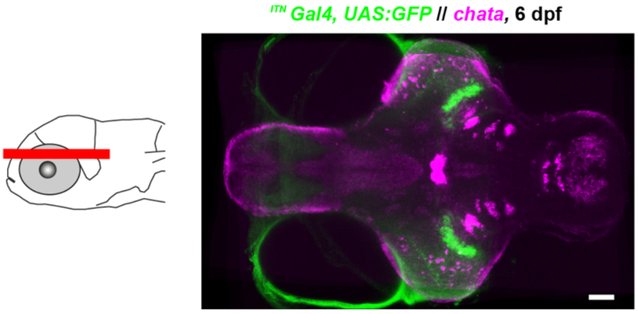

Supplementary Fig. 3

Supplementary Fig. 3. ITNs are neither glutamatergic nor cholinergic.

(a) Expression of *vglut2a*/*vglut2b* as markers for glutamatergic neurons and anti-GFP immunoreactivity in the brain of a 4 dpf ^{ITN}*Gal4, UAS:GCaMP3* larvae (shown cryo-section indicated by the red bar in the schematic larva to the left, scale bar = 50 µm). The ITN cell bodies are visible in the GFP channel (left nucleus highlighted by white rectangle which was then magnified and shown in the lower images for each channel, scale bar = 10 µm). GFP and *vglut2a/vglut2b* expression are mainly non-overlapping (ITNs indicated by asterisks in the lower images).

(b) Overlay of average intensity images derived from datasets of whole-brain GFP-immunoreactivity (green) and *chata* expression (magenta) each registered to the z-brain atlas. GABAergic ITN nuclei are located in the mesencephalic tegmentum, anterior to the midbrain-hindbrain boundary and are not overlapping with cholinergic isthmic nuclei in rhombomere 1. Scale bar = 50 µm.

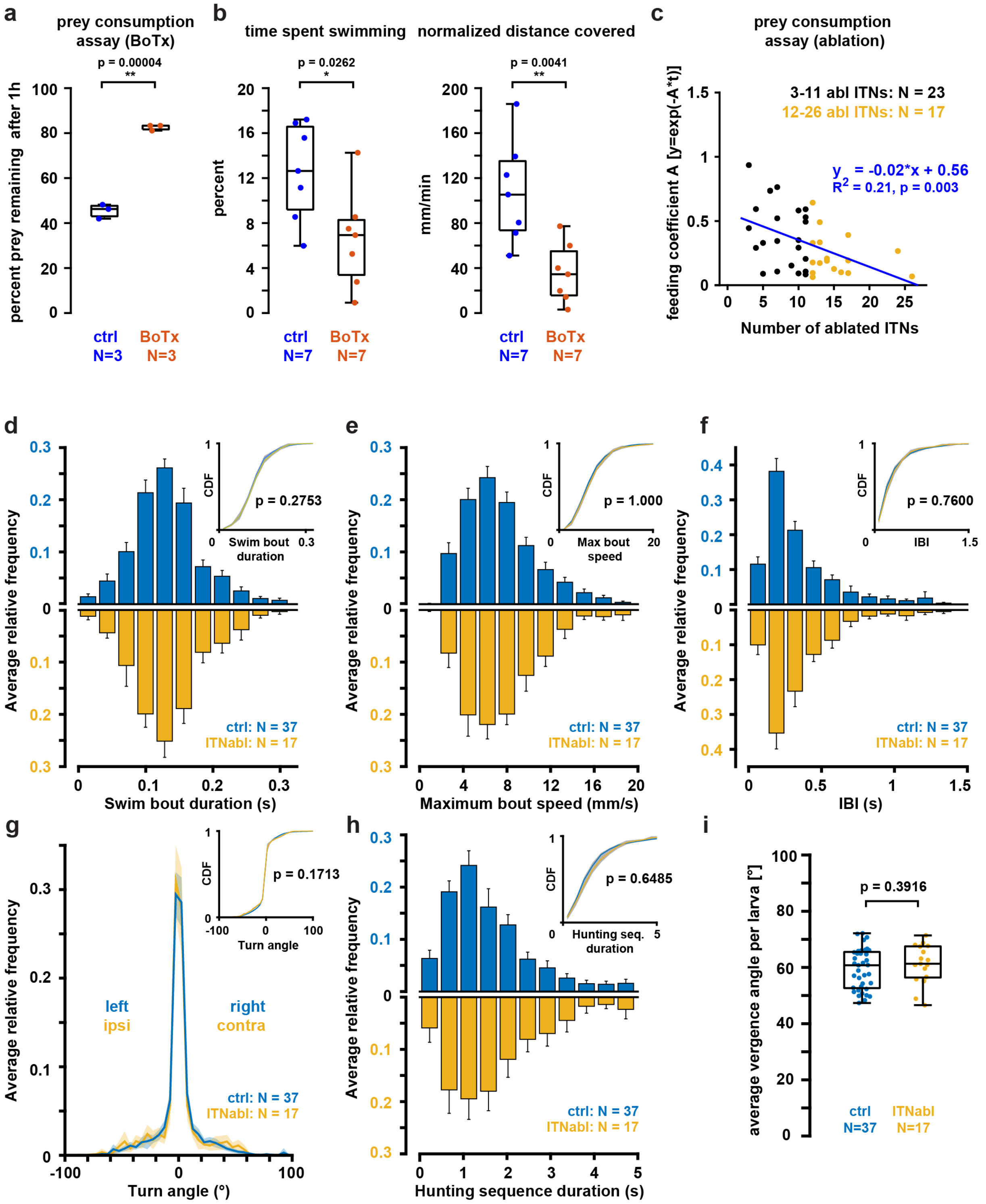

Supplementary Fig. 4

Supplementary Fig. 4. ITN-ablated larvae are indistinguishable from control larvae regarding locomotion and prey recognition.

(a) Silencing of vesicular release in 5 dpf ^{ITN}Gal4 larvae by zebrafish-optimized Botulinum toxin BoTxBLC-GFP (BoTx) caused a severe reduction in prey consumption compared to control siblings in a prey consumption assay (control: median percent prey left after 1h = 46.25 %, BoTx: median percent prey left after 1h = 83.3 %, Mann-Whitney U-test, p = 0.00004, both groups: N = 3 trials, 4 larvae each).

(b) Individual BoTx-expressing larvae spent significantly less time swimming (Mann-Whitney U-test, p = 0.0262) and covered less distance (Mann-Whitney U-test, p = 0.0041) during experimental trials than control siblings (both groups: N = 7 larvae).

(c) The number of ablated ITNs plotted against the feeding coefficient A, derived from temporal feeding curves in the prey consumption assays [y = exp(-A*t)].

(d) Average relative frequencies of swim bout duration during hunting sequences for control (blue) and ITN-ablated larvae (yellow). No significant difference of swim bout duration distributions was found between control and ITN-ablated larvae (control: n = 6749 swim bouts from 37 larvae, median duration = 0.138 s, ITN-ablated: n = 2519 swim bouts from n = 17 larvae, median duration = 0.135 s, Kolmogorov-Smirnov-test, p = 0.2753). Error bars of all following relative frequency plots indicate the +95 % confidence interval. The insets show the cumulative distribution function for control and ITN-ablated histograms with the grey shaded boundaries indicating the 95 % confidence intervals.

(e) Average relative frequencies of maximum swim bout speed during hunting sequences for control (blue) and ITN-ablated larvae (yellow). No significant difference of maximum swim bout speed distributions was found between control and ITN-ablated larvae (control: n = 6749 swim bouts from 37 larvae, median maximum speed = 7.123 mm/s, ITN-ablated: n = 2519 swim bouts from n = 17 larvae, median maximum speed = 7.734 mm/s, Kolmogorov-Smirnov-test, p = 1.000).

(f) Average relative frequencies of interbout interval (IBI) duration during hunting sequences for control (blue) and ITN-ablated larvae (yellow). No significant difference of IBI duration distributions was found between control and ITN-ablated larvae (control: n = 4655 interbout intervals from 37 larvae, median duration = 0.338 s, ITN-ablated: n = 1649 interbout intervals from n = 17 larvae, median duration = 0.339 s, Kolmogorov-Smirnov-test, p = 0.7600).

(g) Average relative frequencies of changes in orientation per swim bout (= turn angle) during hunting sequences with the shaded boundaries indicating the respective 95 % confidence intervals. No difference of swim bout angle distributions was observed between control and ITN-ablated larvae (control: n = 6749 swim bouts / turns from 37 larvae, ITN-ablated: n = 2519 swim bouts / turns from n = 17 larvae, Kolmogorov-Smirnov-test, p = 0.1713).

(h) Average relative frequencies of hunting sequence duration for control (blue) and ITN-ablated larvae (yellow). No significant difference of hunting sequence duration distributions was found between control and ITN-ablated larvae (control: n = 2263 hunting sequences from 37 larvae, median duration = 1.689 s, ITN-ablated: n = 948 hunting sequences from n = 17 larvae, median duration = 1.722 s, Kolmogorov-Smirnov-test, p = 0.6485).

(i) ITN-ablated larvae do not show significantly different average vergence angles during hunting compared to control larvae (control: n = 2263 hunting sequences from 37 larvae, median vergence angle = 60.8 °, ITN-ablated: n = 948 hunting sequences from n = 17 larvae, median vergence angle = 61.3 °, Mann-Whitney U-test, p = 0.3916).

Source data for all panels in this figure are provided as a Source Data file.

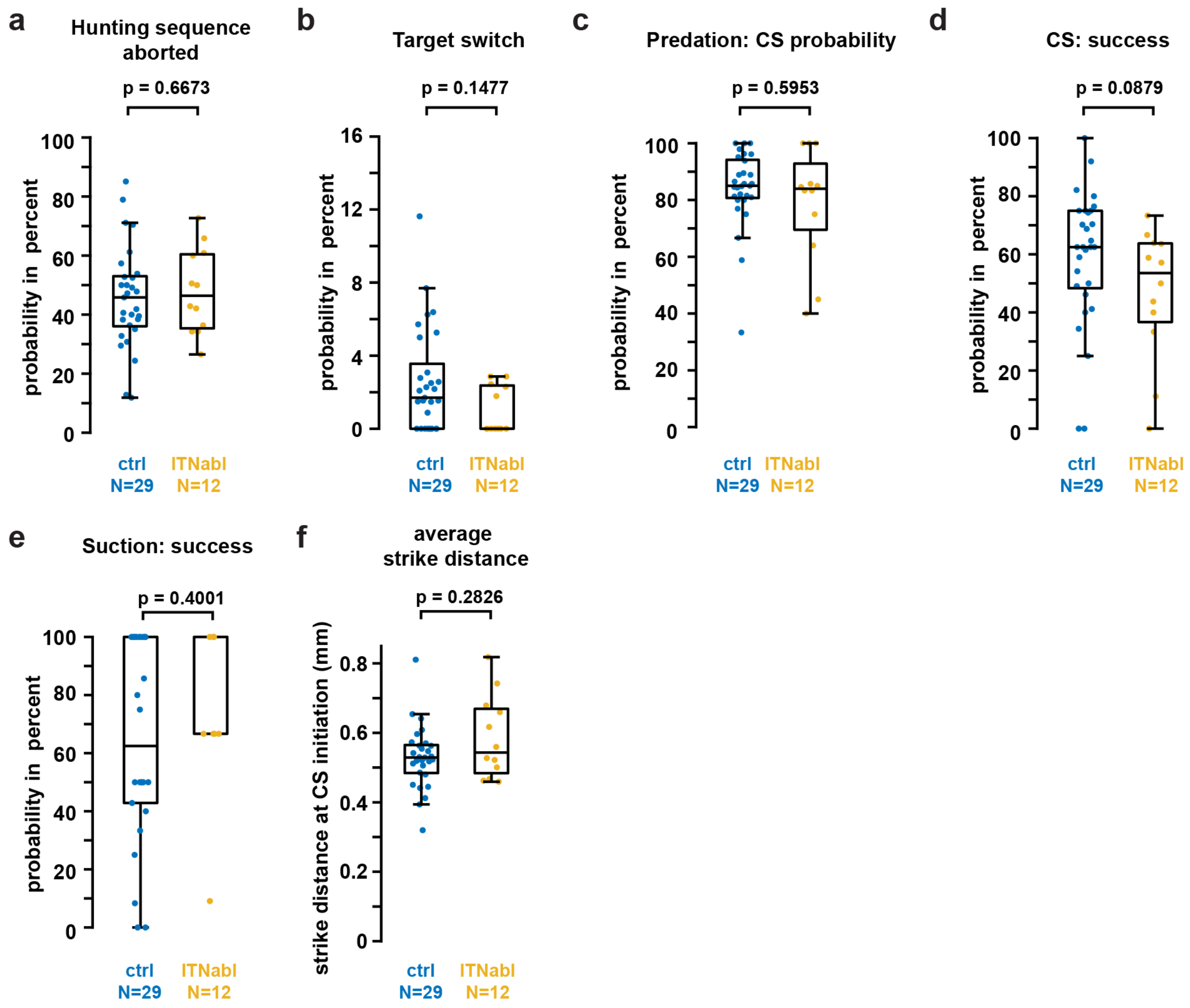

Supplementary Fig. 5

Supplementary Fig. 5. Hunting sequence classification.

(a) ITN-ablated larvae did not abort hunting sequences while the target was outside the binocular strike zone (d > 0.5 mm and/or abs(azimuth) > 10 °) with a different probability compared to control larvae (control: median probability = 45.8 %, ITN-ablated: median probability = 46.4 %, Mann-Whitney U-test, p = 0.6673).

(b) ITN-ablated larvae also did not show a significantly different probability to switch, once one prey target was being pursued, to another target compared to control larvae (control: median target switch probability = 1.7 %, ITN-ablated: median target switch probability = 0 %, Mann-Whitney U-test, p = 0.1477).

(c) If they engaged in predation once targeted prey was in the binocular striking zone, ITN-ablated larvae did not perform capture swims (CS) with a different probability compared to control larvae. This suggests that ITN-ablated larvae did not change their hunting strategy, e.g. by performing more suctions (control: median CS initiation probability = 85.0 %, ITN-ablated: median CS initiation = 84.0 %, Mann-Whitney U-test, p = 0.5953).

(d) ITN-ablated larvae did show a slightly decreased, although not significantly different, probability of successful capture swims (CS) compared to control larvae (control: median probability = 62.5 %, ITN-ablated: median probability = 53.6 %, Mann-Whitney U-test, p = 0.0879).

(e) ITN-ablated larvae did not show a significant different probability of successful suctions compared to control larvae (control: median probability = 62.5 %, ITN-ablated: median probability = 66.6 %, Mann-Whitney U-test, p = 0.4001).

(f) ITN-ablated larvae did not show significantly different average striking distances per larva during capture swims (CS) compared to control larvae (control: median distance = 0.529 mm, ITN-ablated: median distance = 0.543 mm, Mann-Whitney U-test, p = 0.2826). Source data for all panels in this figure are provided as a Source Data file.
