# Supplementary material for: An interhemispheric neural circuit allowing binocular integration in the optic tectum
Source: Nat Commun. 2019 Nov 29;10:5471. doi: 10.1038/s41467-019-13484-9 (PMC6884480; doi:10.1038/s41467-019-13484-9)
Supplement: Supplementary file 3 — Description of Additional Supplementary Files [file 41467_2019_13484_MOESM3_ESM.pdf]

Description of Additional Supplementary Files

File Name: Supplementary Movie 1
Description: Successful capture swim of an unablated control larva at 6 dpf (movie slowed down 10x).

File Name: Supplementary Movie 2
Description: Failure to initiate a capture swim when prey was inside binocular strike zone (d<0.5mm, abs(theta)<10°) in a 6 dpf larva in which 12 ITNs were ablated in the right ITN nucleus (movie slowed down 10x).
