# Supplementary material for: An interhemispheric neural circuit allowing binocular integration in the optic tectum
Source: Nat Commun. 2019 Nov 29;10:5471. doi: 10.1038/s41467-019-13484-9 (PMC6884480; doi:10.1038/s41467-019-13484-9)
Supplement: Supplementary file 6 — Reporting Summary [file 41467_2019_13484_MOESM6_ESM.pdf]

nature research

Corresponding author(s): Filippo Del Bene & Isaac H. Bianco

Last updated by author(s): Oct 20, 2019

Reporting Summary

Nature Research wishes to improve the reproducibility of the work that we publish. This form provides structure for consistency and transparency in reporting. For further information on Nature Research policies, see Authors & Referees and the Editorial Policy Checklist.

Statistics

For all statistical analyses, confirm that the following items are present in the figure legend, table legend, main text, or Methods section.

n/a	Confirmed	
☐	☒	The exact sample size (*n*) for each experimental group/condition, given as a discrete number and unit of measurement
☐	☒	A statement on whether measurements were taken from distinct samples or whether the same sample was measured repeatedly
☐	☒	The statistical test(s) used AND whether they are one- or two-sided<br>*Only common tests should be described solely by name; describe more complex techniques in the Methods section.*
☒	☐	A description of all covariates tested
☒	☐	A description of any assumptions or corrections, such as tests of normality and adjustment for multiple comparisons
☐	☒	A full description of the statistical parameters including central tendency (e.g. means) or other basic estimates (e.g. regression coefficient) AND variation (e.g. standard deviation) or associated estimates of uncertainty (e.g. confidence intervals)
☐	☒	For null hypothesis testing, the test statistic (e.g. *F*, *t*, *r*) with confidence intervals, effect sizes, degrees of freedom and *P* value noted<br>*Give P values as exact values whenever suitable.*
☒	☐	For Bayesian analysis, information on the choice of priors and Markov chain Monte Carlo settings
☒	☐	For hierarchical and complex designs, identification of the appropriate level for tests and full reporting of outcomes
☒	☐	Estimates of effect sizes (e.g. Cohen's *d*, Pearson's *r*), indicating how they were calculated

Our web collection on statistics for biologists contains articles on many of the points above.

Software and code

Policy information about availability of computer code

Data collection	Bonsai 2.0, PsychophysicsToolbox 3.0.13, Matlab 2015a, Labview 2015, ImSpector (Lavision Biotec)
Data analysis	Matlab 2015a, FIJI, Vaa3D

For manuscripts utilizing custom algorithms or software that are central to the research but not yet described in published literature, software must be made available to editors/reviewers. We strongly encourage code deposition in a community repository (e.g. GitHub). See the Nature Research guidelines for submitting code & software for further information.

Data

Policy information about availability of data

All manuscripts must include a data availability statement. This statement should provide the following information, where applicable:

- Accession codes, unique identifiers, or web links for publicly available datasets
- A list of figures that have associated raw data
- A description of any restrictions on data availability

The datasets generated and analyzed during the current study are available from the corresponding authors on request.

Field-specific reporting

Please select the one below that is the best fit for your research. If you are not sure, read the appropriate sections before making your selection.

☒ Life sciences ☐ Behavioural & social sciences ☐ Ecological, evolutionary & environmental sciences

For a reference copy of the document with all sections, see nature.com/documents/nr-reporting-summary-flat.pdf

Life sciences study design

All studies must disclose on these points even when the disclosure is negative.

Sample size	No statistical methods were used to predetermine sample size.
Data exclusions	Larvae that did not show visible heartbeat and bloodflow in both brain hemispheres or that did not survive the ablation procedure were excluded from behavioral testing.
Replication	For calcium imaging, ablation and behavioral analysis of prey hunting behavior one individual larva was considered an independent experiment replication. For transient plasmid expression, injection of embryos from a clutch laid by one couple of adult zebrafish was considered as one independent experiment replication For immunohistochemistry, one sectioned and stained fish represents one independent experiment.
Randomization	Samples were not randomized as allocation of animals to groups (e.g. ctrl, ablated, sham-ablated) was entirely based on the intervention type.
Blinding	Control or ablated larvae were individually separated into 12-well plates and assigned a code for anonymization. As there were no external differences between control and ablated larvae the investigators were blind to the identity of the animal during the analysis.

Reporting for specific materials, systems and methods

We require information from authors about some types of materials, experimental systems and methods used in many studies. Here, indicate whether each material, system or method listed is relevant to your study. If you are not sure if a list item applies to your research, read the appropriate section before selecting a response.

Materials & experimental systems

n/a	Involved in the study	
☐	☒	Antibodies
☒	☐	Eukaryotic cell lines
☒	☐	Palaeontology
☐	☒	Animals and other organisms
☒	☐	Human research participants
☒	☐	Clinical data

Methods

n/a	Involved in the study	
☒	☐	ChIP-seq
☒	☐	Flow cytometry
☒	☐	MRI-based neuroimaging

Antibodies

Antibodies used	chicken anti-GFP antibody (Genetex, GTX13970); AlexaFluor488 goat anti-chick secondary antibody (Invitrogen, A11039)
Validation	http://www.genetex.com/GFP-antibody-GTX13970.html

Animals and other organisms

Policy information about studies involving animals; ARRIVE guidelines recommended for reporting animal research

Laboratory animals	zebrafish in TL or AB background. Larval zebrafish were studied before the onset of sexual differentiation.
Wild animals	This study did not involve wild animals
Field-collected samples	This study did not involve samples collected from the field.
Ethics oversight	All animal procedures were performed in accordance with French, British and European Union animal welfare guidelines. Animal handling and experimental procedures were approved by the Committee on ethics of animal experimentation - Institut Curie and the UK Home Office under the Animal (Scientific Procedures) Act 1986.

Note that full information on the approval of the study protocol must also be provided in the manuscript.
